# Characterization of novel microneme adhesive repeats (MAR) in *Eimeria tenella*

**DOI:** 10.1186/s13071-017-2454-4

**Published:** 2017-10-17

**Authors:** Virginia Marugan-Hernandez, Rebekah Fiddy, Jazmine Nurse-Francis, Oliver Smith, Laura Pritchard, Fiona M. Tomley

**Affiliations:** Department of Pathobiology and Population Sciences, The Royal Veterinary College, University of London, Hawkshead Lane, North Mymms, Hatfield, Hertfordshire, AL9 7TA UK

**Keywords:** *Eimeria tenella*, Microneme proteins, Binding domains, Parasite-host interaction

## Abstract

**Background:**

The phylum Apicomplexa comprises a wide variety of parasites of significant medical and economic relevance. These parasites have extremely different host and tissue tropisms; for example *Toxoplasma gondii* can invade virtually any nucleated cell and infect almost all warm-blooded vertebrates, whereas *Eimeria tenella* infects only chickens and is restricted in its growth to epithelial cells of the caecum. Proteins released from the microneme secretory organelles (MICs) are critical for apicomplexan invasion of host cells and allow parasites to bind a diverse range of host cell oligosaccharide epitopes. MICs bear modular arrangements of sequences with adhesive proteins and interestingly the sialic-acid binding MAR (microneme adhesive repeat) domain containing proteins (MCPs) are suggested to make significant contributions to the different host and tissue tropisms of *T. gondii* and *E. tenella*.

**Results:**

In this study, we evaluated the binding capacity of Type I MAR domains from novel *E. tenella* MCPs. Variants of the previously described HxT motif were analysed showing that HxT and VxT variants bind, whereas HxS and YxE variants did not. One of these MCP containing a single MAR (EtMCP2) showed an apical localization when expressed as a fusion with the fluorescent reporter mCherry in transgenic populations and a similar pattern of transcripts per zoite during endogenous development in vitro as the well-characterised microneme protein EtMIC2.

**Conclusions:**

Variation in the binding properties of the MAR of different EtMCPs was confirmed and their ability to bind a wider range of sialic acids and terminal linkages should be studied. In addition, transgenesis technology has been used for first time in *Eimeria* parasites as a rapid tool for the study of endogenous protein localization by fusion with a fluorescent reporter.

**Electronic supplementary material:**

The online version of this article (10.1186/s13071-017-2454-4) contains supplementary material, which is available to authorized users.

## Background

Seven species of the genus *Eimeria* (Apicomplexa, Coccidia) cause chicken coccidiosis, a disease with a huge economic impact in the poultry industry. Disease pathology is characterised by diarrhoea, malabsorption and for some species haemorrhage, and has a severe impact on animal welfare, efficiency of feed conversion and weight gain. *Eimeria* parasites disseminate readily through flocks via the oral-faecal route and are highly prevalent throughout the world [1]. Coccidiosis control relies mainly on in-feed anticoccidial drugs; however, drug resistance is ubiquitous and there are increasing concerns from consumers regarding risks of drug residues entering the food chain. Immune-prophylaxis using live-attenuated vaccines is also effective for coccidiosis control and is widely used in breeding and laying flocks. However, because the parasites do not replicate outside of their host, vaccine production requires large numbers of chickens to amplify lines of vaccinal parasites; this places a practical limitation on production and means that vaccines are costly compared to anticoccidial drugs [2].

Understanding the biology of *Eimeria* parasites in order to elucidate the functional significance and immunogenicity of parasite sub-cellular structures aids discovery of antigens for new types of vaccines. A substantial amount of data from high-throughput technologies is now available [3–5]. However, there is a clear imbalance between these new data and our capacity to validate the role of specific proteins in key biological processes. Conventional methods for protein characterisation are time-consuming, expensive and not always conclusive. Complementary technologies based on reverse genetics are available for some apicomplexan parasites, including the coccidian *Toxoplasma gondii* [6], and are important tools to study gene functionality. For example, gene knock-in and knock-out has been used to investigate parasite invasion and protein trafficking [7, 8] and to elucidate the functional role of several vaccine antigens including TgAMA1 and TgROP18 [9, 10]. Reverse genetics tools for *Eimeria* spp. are less developed, however, efficient random integration of transgenes is well established [11, 12] for expressing and targeting of reporter molecules and heterologous antigens [13–17].

Proteins secreted from apicomplexan microneme organelles (MICs) play important roles in parasite adhesion and invasion of host cells [18]. Some MIC proteins are specific for coccidial parasites and mediate parasite recognition of host cell molecules. These include a family of MICs that contain copies of a protein domain termed microneme adhesive repeat (MAR) that binds sialic acid [19, 20]. Binding to the sialyl ligand is co-ordinated through hydrogen bonding and pi-stacking to histidine and threonine residues in a HxT motif that is conserved across many, but not all, MAR [19, 21]. All MAR domains adopt the same structural fold, but they are subclassified into type I and type II MAR on the basis of their primary sequence, with type I having an extended ɑ1helix/loop region and (some) type II having an additional C-terminal β-finger region [21]. These subtle structural differences, in addition to specific sequence divergence within individual MAR, confer differential binding properties. In particular, bulky side chains in the extended ɑ1helix/loop region of Type I MAR (specifically a LxxY motif in MAR from the *E. tenella* protein EtMIC3) make increased contacts to the sialyl saccharide leading to a higher level of specificity in binding, and precluding interaction with N-glycolyl sialic acids [22].

*Toxoplasma gondii* and *Neospora caninum* possess proteins with both Type I and Type II MAR. However, to date all MAR domains identified in *Eimeria* species are exclusively Type I, suggesting that a lack of Type II MAR may contribute to the exquisite host and site specificity displayed by these parasites [21, 22]. For *Eimeria tenella*, EtMIC3 containing seven Type I MAR binds a restricted range of sialyl glycans with a strong preference for ɑ2,3-linkages that are predominant in the chicken gut [22, 23]. Four additional Type I MAR containing proteins (EtMCP2 with 1 MAR; EtMCP3 with 3 MAR; EtMCP4 with 4 MAR, EtMCP5 with 2 MAR) are present in the *E. tenella* genome [21] (Table 1) but their subcellular location, stage-expression and function are unknown. Whilst five of the seven Type I MAR of EtMIC3 have the conserved sialic acid binding motif HxT, this is present in only two of the ten Type I MAR of the EtMCPs, with the remainder having partial or no conservation of the motif.

**Table 1:** Summary of *E. tenella* MCPs and their binding properties

Repeat	Accession number	Motif	Recombinant expression	Fetuin / MDBK binding
EtMCP2.1	ETH_00020995	H – L – S	recMCP2.1	No
EtMCP3.1	ETH_00030520	H – W – S	–	–
EtMCP3.2		V – W – T	–	–
EtMCP3.3		H – W – T	recMCP3.3	Yes
EtMCP4.1	ETH_00030525	V – W – S	–	–
EtMCP4.2		H – W – S	recMCP4.2	No
EtMCP4.3		V – W – T	recMCP4.3	Yes
EtMCP4.4		H – W – T	–	–
EtMCP5.1	ETH_00003280	X – X – X^a^	–	–
EtMCP5.2		X – X – X^a^	recMCP5.2	No
EtMIC3.1	ETH_00021010	Y – L – T	–	–
EtMIC3.2		H – L – T	–	–
EtMIC3.3		H – L – T	recMIC3.3	Yes
EtMIC3.4		H – L – T	–	–
EtMIC3.5		H – L – T	–	–
EtMIC3.6		H – L – T	–	–
EtMIC3.7		X – X – X^a^	–	–

^a^X – X – X = motif not identifiable

In this paper, we examine the ability of EtMCP Type I MARs with variant HxT motifs to bind in vitro to fetuin-agarose or to cultured cells. To explore the subcellular location of an MCP with a variant, non-binding motif (EtMCP2), we generated a transgenic population of *E. tenella* parasites that overexpresses EtMCP2 as a mCherry fusion. We show that this fusion localises to the apical tip of the sporozoites, in a pattern similar to that of a transgenic population overexpressing mCherry fused to the known microneme protein EtMIC2. Furthermore, using wild type parasites we show by quantitative PCR (qPCR) and reverse-transcription qPCR (RT-qPCR) that EtMCP2 transcript/genome ratio during sporozoite invasion and intracellular schizogony is similar to that of the known microneme proteins, EtMIC2 and EtMIC5. Thus, despite an inability to bind sialyl moieties, the Type I MAR protein, EtMCP2, appears to be expressed within the microneme organelles of *E. tenella*.

## Methods

### Parasites and birds

*Eimeria tenella* Wisconsin strain [24] was propagated in vivo in 3 week old Lohmann White chickens obtained from APHA Weybridge, where they were produced and reared under specific pathogen free conditions. Oocyst harvesting and cleaning [25], and sporozoite excystation and purification [26] were carried out as previously described. For in vitro cell culture assays, purified sporozoites (wild type or transgenic) were added to confluent MDBK cell monolayers in 24-well plates (0.3 × 10^6^ cells/well) at a multiplicity of infection of 1:1 and incubated at 41 °C / 5% CO_2_ for 4, 24, 48 and 72 h.

### Expression and purification of recombinant MAR regions in *E. coli*

Complementary DNA (cDNA) sequences corresponding to protein repeats containing MAR with varying HxT motifs (Table 1) were amplified from parasite cDNA using the primers listed in Additional file 1: Table S1 and cloned between *Nco*I and *Not*I restriction sites into the plasmid pET32b (+) (Novagen, Hertfordshire, UK). The recombinant proteins were expressed as soluble polyhistidine (His6) and thioredoxin fusions in BL21 (DE3) pLysS *E. coli* (Stratagene, California, USA) and were purified and quantified as described previously [17].

### Fetuin-agarose affinity binding assay

Fetuin agarose saline suspension (500 μl, Sigma-Aldrich, Suffolk, UK) was packed in mini filter columns and equilibrated with phosphate buffer (100 mM, pH 6.8). Recombinant protein quantified by Bradford (Sigma-Aldrich) was suspended in phosphate buffer (200 μg), loaded to the top of the affinity column and maintained at 4 °C for 3 h for complete binding [27]. The columns were washed with phosphate buffer containing NaCl (100 mM) to remove non-specifically bound proteins. The fetuin-binding fraction was then eluted from the column in sodium citrate (0.1 M, pH 6.7) and kept at -20 °C for further analysis.

### MDBK cell binding assay

MDBK cells were seeded into 24-well plates (0.3 × 10^6^ cells/well) and left to settle into a monolayer for 4 h, washed gently in phosphate buffered saline (PBS), blocked in 1% bovine serum albumin (BSA) for 30 min, and washed again in PBS. 500 μg of recombinant protein was added per well and incubated for 1 h at 4 °C. Supernatants were removed and the cells were washed several times with PBS. MDBK monolayers were removed from wells with trypsin (Sigma-Aldrich), pelleted and stored at -20 °C until further analysis.

### SDS-polyacrylamide gel electrophoresis (PAGE) and western blot

Eluted fetuin-binding fraction (50 μg in 50 μg of 2× Laemmli loading buffer (Sigma-Aldrich)) and MDBK-bound protein fractions (resuspended in 100 μg 1× Laemmli) were electrophoresed in NuPAGE 4–12% Bis-Tris 10-well gels (Invitrogen, California, USA) and stained with Coomassie blue (BioRad, Hertfordshire, UK). Proteins were transferred from gels to nitrocellulose membranes (GE Healthcare, Buckinghamshire, UK) by semi-dry blotting following the manufacturer’s protocols (Invitrogen), and membranes were blocked in 5% (*w*/*v*) non-fat milk (Bio-Rad) overnight. Membranes were incubated for 1 h with mouse anti-histidine tag antibody (1/1000; Merck Millipore, Hertfordshire, UK), then washed three times with tris buffered saline-Tween20 (TBS-Tween) 0.05% (*v*/v) and incubated for 1 h with goat anti-mouse IgG antibody HRP conjugate (1/5000; Merck Millipore). Membranes were washed three times with TBS-Tween 0.05% (v/v), once with TBS and finally with distilled water before adding Luminata substrate (Merck Millipore). Chemiluminiscence was visualised in a G:BOX (Syngene, Cambridge, UK) and images were taken with GeneSnap 7.12 software (Syngene).

### Plasmid construct for the expression of EtMCP2-mCherry fusion in *E. tenella*

The plasmid pMIC2-mChe [28] containing the complete coding sequence of microneme protein EtMIC2 fused to the fluorescent reporter mCherry was the starting plasmid used to generate a further plasmid containing the coding sequence of EtMCP2 fused to mCherry (pMCP2-mChe). Both plasmids contain a second expression cassette that includes the coding sequence of mCitrine for the detection and sorting by FACS of transgenic parasites. Briefly, *EtMCP2* sequence lacking start and stop codons was PCR-amplified (see primers in Additional file 1: Table S1) and cloned into *XbaI* restriction sites in pMIC2-mChe, directly replacing *EtMIC2* coding sequence but retaining *EtMIC2* start codon. The PCR, sequencing, molecular cloning and plasmid preparation for transfection were carried out as described previously [17].

### Generation of transgenic populations of *E. tenella* expressing mCherry fusions

Restriction enzyme-mediated integration (REMI) transfection into *E. tenella* sporozoites was carried out using programme EO114 of the Nucleofector 4D (Lonza®, Basel, Switzerland) following protocols described previously [17]. After 24 h of incubation with MDBK cells, the presence of transiently transfected sporozoites expressing fluorescent reporters mCitrine and mCherry was confirmed by fluorescence microscopy (Leica Microsystems DMI3000B). To obtain stable transgenic populations (EtMIC2-mChe and EtMCP2-mChe), 0.75 × 10^6^ transfected sporozoites were used to infect two chickens via the cloacal route. Shed fluorescent oocysts were selected by FACS (BD FACS Aria™ III) for subsequent in vivo passages to amplify the proportion of transgenic parasites within the populations.

Infected monolayers and oocysts of transgenic populations were observed under UV fluorescence in a Leica DMI3000B microscope and photographed with a Leica DCF365FX camera. Image processing was performed using the LAS AF (Leica Microsystems).

### Immunofluorescence

MDBK monolayers infected for 4 h with *E. tenella* sporozoites were fixed in 4% paraformaldehyde in PBS for 15 min and washed in PBS. Fixed monolayers were blocked by incubating in 3% BSA, 0.25% Triton ×100 in PBS for 30 min then in rabbit anti-EtMIC2 serum (1/500) for 1 h. After three washes in PBS, monolayers were incubated with goat anti-rabbit IgG conjugated to Alexa Fluor 488 (1/1000; Invitrogen), washed in PBS, then observed under UV fluorescence using a Leica DMI3000B microscope and photographed with a Leica DCF365FX camera. Image processing was performed using the LAS AF (Leica Microsystems).

### Real time quantitative PCR (qPCR) and reverse transcription qPCR (RT-qPCR)

Total DNA and RNA were extracted from an in vitro time course of parasite development in MDBK cells infected with wild-type *E. tenella* sporozoites following the manufacturer’s protocols (All prep DNA/RNA Mini Kit, Qiagen, West Sussex, UK). cDNA was synthesized from total RNA using SuperScript II® reverse transcriptase (Invitrogen) as described previously [17].

Real time qPCR was performed in a CFX96 Touch® Real-Time PCR Detection System (Bio-Rad) using DNA-binding dye SsoFastTM EvaGreen® Supermix (Bio-Rad) as described previously [17]. The number of haploid genomes (equivalent to sporozoites or merozoites) per well was determined for each time point using gDNA specific primers for *Eimeria* spp. 5S rDNA [11] and a standard curve of sporozoite gDNA extracted by the same methods. Transgene transcription was quantified from cDNA using specific primers for *EtMIC2*, *EtMIC5*, *EtMCP2* and *EtActin* (Additional file 1: Table S1) and compared with serial dilutions of DNA standard templates for each transgene (pGEM®-T Easy (Promega, Hampshire, UK) plasmid containing the *EtMIC2*, *EtMIC5*, *EtMCP2* or *EtActin* coding sequences). Standard curves were prepared for all genes from 10^7^ to 10^2^ sporozoites or plasmid copies, against which the total number of parasites or transcript copy numbers were quantified, respectively. The transcription levels along the course of intracellular schizont development were normalised to the number of parasite genomes. Data were analysed by a one-way ANOVA with post-hoc Bonferroni test (SPSS Statistics 22, New York, USA).

## Results

### Type I MAR from *E. tenella* MCPs has low conservation in their binding domains

**Fig. 1:**
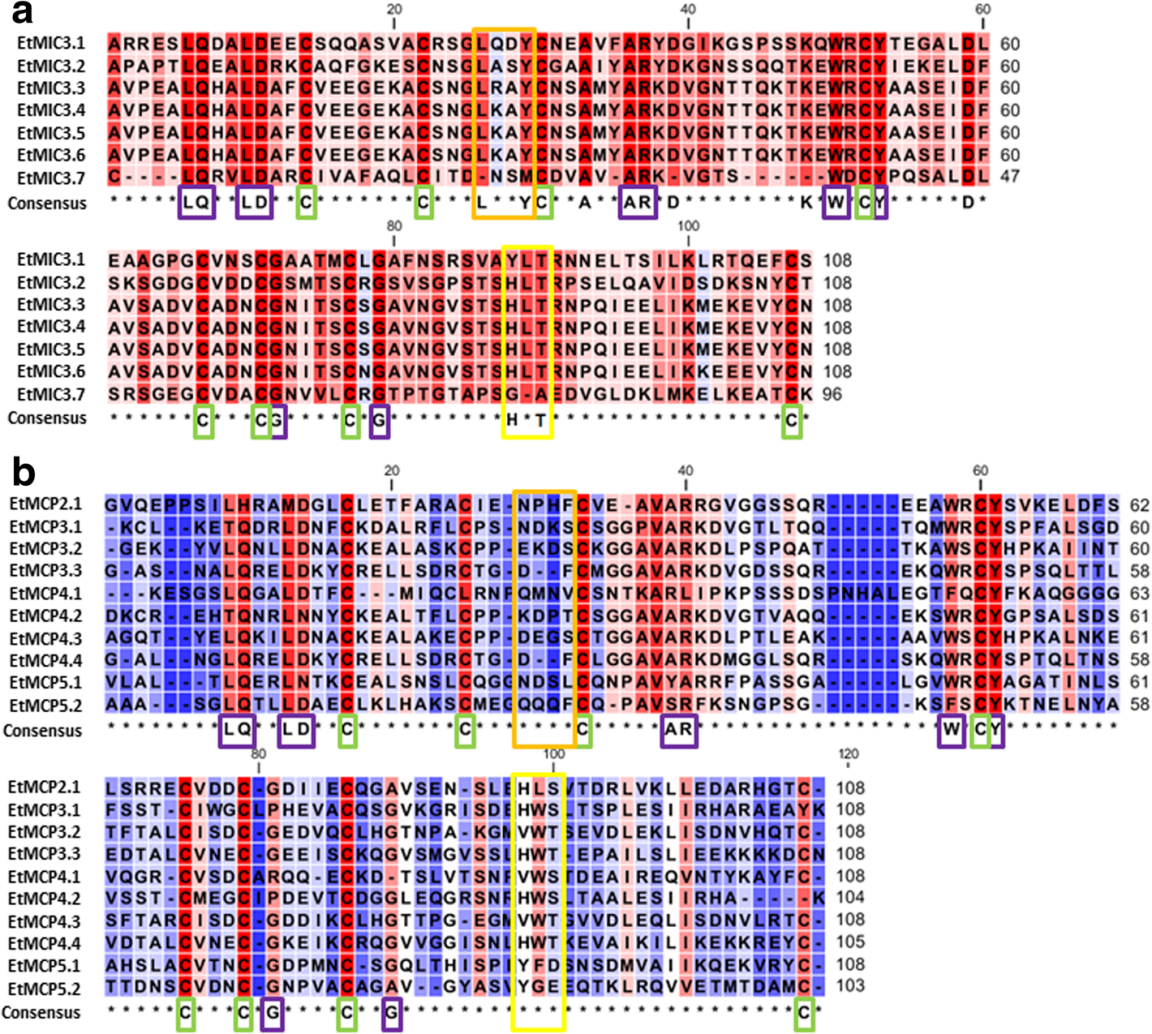
Alignment of MAR domains of EtMIC3 (7 repeats) (**a**) and four EtMCPs (MCP2, 1 repeat; MCP3, 3 repeats; MCP4, 4 repeats; MCP5, 2 repeats) (**b**). High/low similarity is represented by different intensities of red/blue, respectively. Green boxes indicate conserved cysteines (C); purple boxes indicate conserved residues; orange boxes indicate LxxY residue within the ɑ-helix/loop extension of MAR type I; yellow boxes indicate HxT/HxS residue coordinating binding to the sialic acid saccharides

MAR from EtMIC3 (Fig. 1a) and from the four identified EtMCP proteins (Fig. 1b) of *E. tenella* were aligned in order to predict their potential binding properties (Table 1). All MAR from EtMIC3 contain eight cysteine (C) residues (Fig. 1a; green boxes) as do most MAR from EtMCPs except for EtMCP3.1 and EtMCP4.2 which lack the last one (Fig. 1b; green boxes). Most of the conserved signatures described by Blumenschein et al. [19] and Lai et al. [22] in Type I and Type II MAR are present in at least 70% of the aligned Type I MAR from EtMCPs (Fig. 1b; purple boxes). However, the LxxY signature within the ɑ1-helix/loop extension of Type I MAR (Fig. 1a; orange boxes) that is proposed to restrict interaction with some sialyl saccharides [22] is not conserved in any MAR from the EtMCPs (Fig. 1b; orange boxes; Table 2). Moreover, the HxT motif that directly coordinates binding to sialyl saccharides (Fig.1a; yellow box) exhibits considerable variation in EtMCPs (Fig.1b; yellow box) as summarised in Table 1.

### Type I MAR with conserved threonine residues bind to fetuin and to cultured cells

**Fig. 2:**
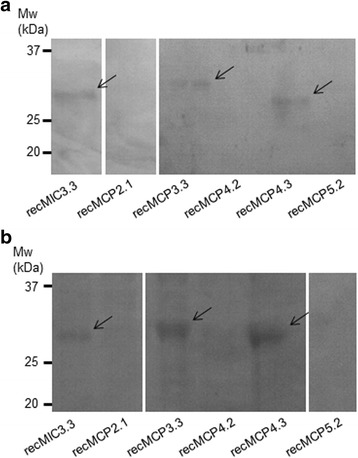
Binding assays of recombinant MAR from diverse EtMCPs. Western blotting of eluted or binding fractions after incubation with fetuin (**a**) or MDBK cells (**b**). Membranes were incubated with anti-histidine tag antibody as primary antibody and goat anti-mouse IgG antibody HRP conjugate was used as secondary antibody. Arrows indicate positive bands

Variant MAR from EtMCPs (recMCP2.1/3.3/4.2/4.3/5.2) as well as MAR3 of EtMIC3 (recMIC3.3) were expressed as soluble recombinant fusion proteins in *E. coli* (Table 1) and tested for their ability to bind highly sialylated fetuin agarose and Madin-Darby bovine kidney (MDBK) cells. All recombinant MAR that retain the threonine residue within the HxT motif that co-ordinates sialyl binding (HLT in recMIC3.3, HWT in recMCP3.3 and VWT in recMCP4.3) bound to both fetuin agarose and MDBK cells (Fig. 2a and b, Lanes 1, 3 and 5, Table 1). All other variants that were tested (HLS in recMCP2.1, HWS in recMCP4.2 and YGE in recMCP5.2) did not bind to either target (Fig. 2a and b, Lanes 2, 4 and 6, Table 1).

### EtMCP2 fused to mCherry localises to the anterior end of *E. tenella* sporozoites in both transient and stable transgenic populations

**Fig. 3:**
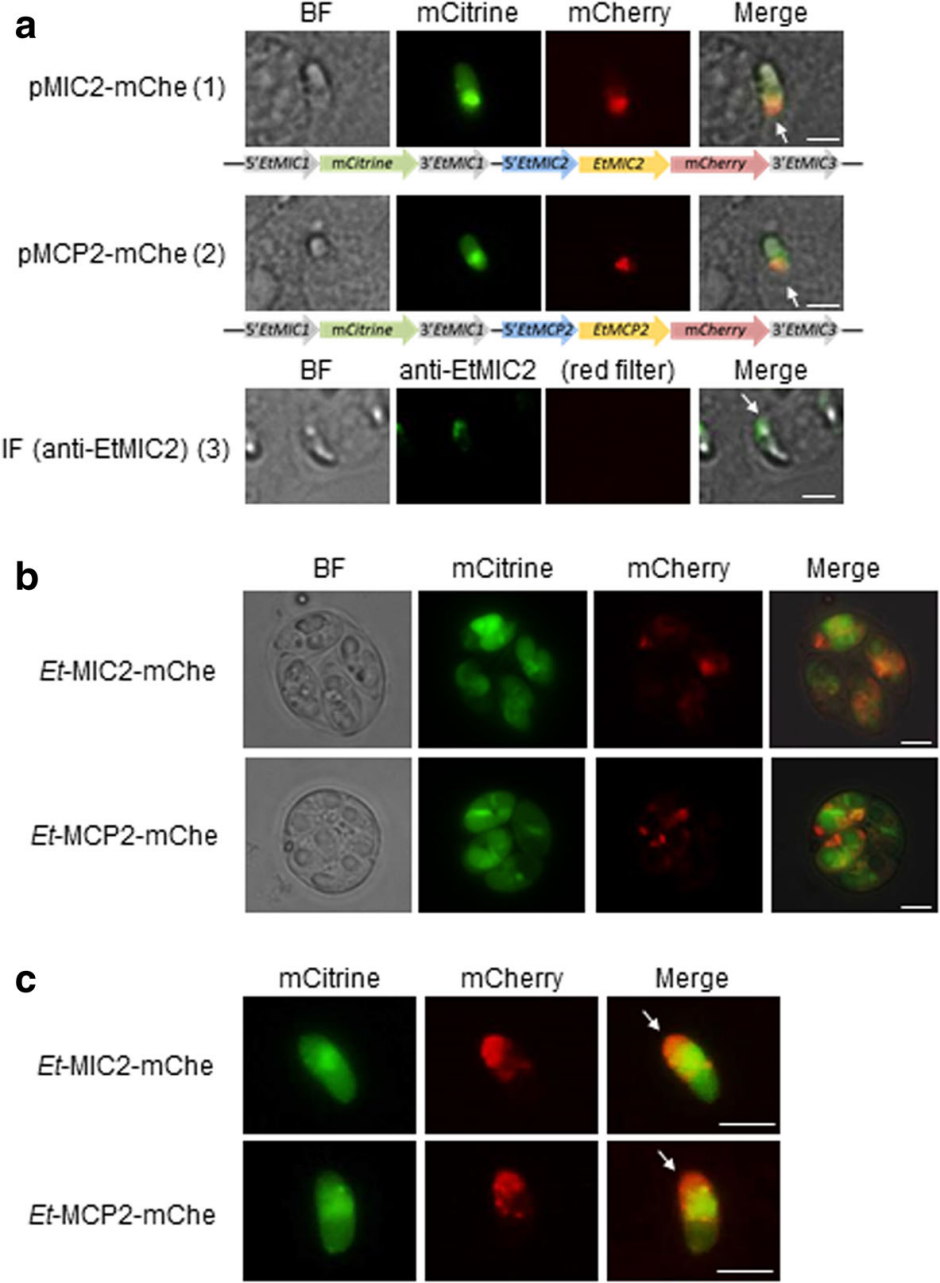
Fluorescence observed in transgenic populations of *E. tenella*. **a** Transiently transfected sporozoites with MDBK cells at 24 h post-infection. Panel 1: pMIC2-mChe transfected sporozoites; mCherry is seen at the apical end whereas mCitrine is detected in the cytosol. Panel 2: pMCP2-mChe transfected sporozoites; mCherry is seen at the apical end part whereas mCitrine is detected in the cytosol. Panel 3: immunofluorescence (IF) of *E. tenella* wild type sporozoites probed with anti-EtMIC2 and goat anti-rabbit IgG conjugated to Alexa Fluor 488. The plasmid constructs for each transfection are displayed above each panel. Arrows indicate the anterior end of sporozoites. **b** Oocysts of stable transgenic populations *Et*-MIC2-mChe and *Et*-MCP2-mChe; mCitrine was observed in the cytosol of encysted sporozoites whereas mCherry accumulated in specific patches (location cannot be assigned due the folded position of the sporozoites within the oocyst). **c** Sporozoites of stable transgenic populations *Et*-MIC2-mChe and *Et*-MCP2-mChe incubated with MDBK cells for 24 h; mCherry was observed at apical end whereas mCitrine was detected the cytosol for both populations. *Scale-bars*: 5 μm

Sporozoites were transfected with plasmids that contain the entire coding sequences (including predicted N-terminal signal peptides) of either *EtMIC2* or *EtMCP2* fused directly to *mCherry* (pMIC2-mChe or pMCP2-mChe; Additional file 1: Figure S1) alongside a second cassette that independently expresses mCitrine. Transiently transfected sporozoites in MDBK cell cultures were found to express both reporters, with green (mCitrine) expression predominantly in the cytosol and red (mCherry) expression restricted to the anterior part of the sporozoite for both pMIC2-mChe and pMCP2-mChe transfectants (Fig. 3a panel 1 and panel 2, respectively). EtMIC2 is a well described microneme protein [29] and its localisation when overexpressed as an mCherry fusion is similar to what is seen by immunofluorescence when MDBK monolayers are infected with wild-type sporozoites and probed with anti-EtMIC2 serum (compare Fig. 3a panel 1 mCherry red fluorescence with panel 3 green Alexa Fluor 488 immunofluorescence). EtMCP2 fused to mCherry shows a similar pattern (Fig. 3a panel 2 mCherry red fluorescence), which allows us to conclude is also likely to be located in the micronemes.

Transfected sporozoites were used to generate stable transgenic populations (*Et*-MIC2-mChe and *Et*-MCP2-mChe) by two consecutive passages through chickens with fluorescence-activated cell sorting (FACS) in between to enrich the populations in fluorescent oocysts. Within oocysts (Fig. 3b) for both populations, mCherry exhibited a different pattern of expression to that seen for cytosolic mCitrine, indicating a non-cytosolic location within the sporozoite; it was not possible to discern the precise location of expression. However, after hatching sporozoites of the transgenic populations and allowing these to invade MDBK cells (Fig. 3c) it could be clearly seen that fluorescence corresponded to what had been observed in transiently transfected sporozoites (Fig. 3a panels 1 and 2) with mCitrine predominantly in the cytosol and mCherry in the apical tip, as expected for microneme proteins. This pattern was similarly observed in freshly excysted sporozoites before they invaded host cells (not shown).

### Abundance of *EtMCP2* transcripts during schizogony and comparison with genes encoding known microneme proteins

**Fig. 4:**
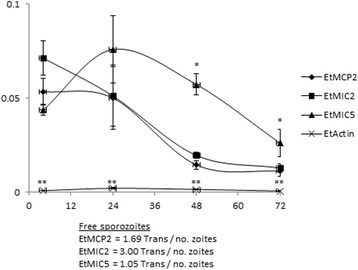
Analysis of transcript abundance of *E. tenella* micromene proteins EtMIC2 and EtMIC5, EtActin and EtMCP2. Transcript number per zoite during a time course of intracellular development of parasites were similar for EtMIC2 and EtMCP2. Asrterisks indicate significant differences (*P < 0.05, **P < 0.01, one-way ANOVA with Bonferroni *post-hoc* test). Transcription levels per zoite obtained in free purified sporozoites before invasion are shown

We documented the transcript numbers per zoite of *EtMCP2* during endogenous parasite development in vitro by RT-qPCR and compared this to transcripts per zoite of *EtMIC2*, *EtMIC5* (encoding two well characterised EtMIC proteins, EtMIC2 and EtMIC5 [29, 30]) and *EtActin* (which is expressed at a low level throughout parasite growth). In free sporozoites, *EtMIC2* transcripts (Fig. 4) were at a significantly higher level than those of *EtMIC5* (*F*_(3,8)_ = 22.708, *P* = 0.012) and slightly higher, but not significantly, than those of *Et*MCP2 (*F*_(3,8)_ = 22.708, *P* = 0.066). As schizogony progressed, values decreased significantly for all three genes. A very similar pattern was observed for *EtMIC2* and *EtMCP2*, where number of transcripts per zoite were markedly reduced during schizogony (Fig. 4, 24–48 hpi), remaining at the same low level once merozoites are formed and released (48–72 hpi). In contrast, *EtMIC5* transcripts per zoite were significantly higher during late endogenous development and formation and release of merozoites (48–72 hpi; *F*_(3,8)_ = 84.105, *P* < 0.0001 and *F*_(3,8)_ = 28.489, *P* = 0.019, respectively) compared to EtMIC2 levels. As expected, *EtActin* was low and constant along the endogenous development.

## Discussion

Microneme proteins are characterised by the possession of adhesive domains that play roles in parasite attachment and invasion of the host cell. One of these, the MAR domain, is restricted to coccidial parasites and has been shown to bind sialylated glycans. Structural and sequence variations between and within Type I and Type II MAR family domains results in variant capacities for binding sialylated residues, contributing to differences in host and site specificities of the parasites. For *E. tenella* the only MAR protein explored to date is microneme protein EtMIC3; this has 7 Type I MAR, is expressed by invading sporozoites, binds the surface of chicken caecal epithelial cells, and has a strong preference for terminal N-acetylneuraminic acids with ɑ2,3-linkages [22]. Several additional genes encoding proteins with Type I MAR domains are annotated in the *E. tenella* genome (EtMCP2 to EtMCP5), however, until now their adhesive properties and subcellular locations were unconfirmed.

In this study, MAR from *E. tenella* MCPs were analysed for their predicted and actual binding properties, along with MAR3 from EtMIC3. Blumenschein et al. [19] reported the involvement of the threonine (T) residue in host cell carbohydrate binding; our results highlight that the threonine residue within the HxT binding domain of MAR seems to be essential for binding to fetuin or to cultured MDBK cells. None of the MAR with threonine substitutions (serine in recMCP2.1 and recMCP4.2, glutamic acid in recMCP5.2) were able to bind. However, substitution of the histidine within the HxT binding domain with a valine in recMCP4.3 was tolerated and this MAR was able to bind both targets. Interestingly, none of the EtMCP MAR domains were found contain the LxxY motif that is present in the MARI extension of the ɑ1-helix/loop of the EtMIC3 MAR. The leucine and tyrosine residues in this motif were structurally implicated in steric hindrance within the binding pocket, resulting in EtMIC3 being able to bind only a restricted range of sialyl glycans [22]. It would be interesting therefore to explore whether the MAR from *E. tenella* MCP are able to bind a wider range of sialic acids, including N-glycolyneuraminic acids, and a wider range of terminal linkages.

We aimed to determine whether EtMCP proteins are localised to the microneme organelles and chose to explore this using EtMCP2, because its small size (encompassing an N-terminal signal peptide plus a single MAR domain) was most convenient for tagging and transfection into *E. tenella*. Expression of EtMCP2 fused at its C-terminus to mCherry resulted in the fused protein being expressed with an apical location within the sporozoite in both transiently transfected and stable transgenic populations. By comparing the pattern of expression to that seen with an analogous mCherry fusion with the characterised microneme protein EtMIC2, it seems highly likely that EtMCP2 is indeed localised to the microneme organelles where it presumably plays a role in binding to host cells.

In addition to the putative micronemal localisation, the accumulation of transcripts per zoites expressed from *EtMCP2* and *EtMIC2* exhibited a similar dynamic pattern during intracellular development of *E. tenella* within cultured MDBK cells. The relative numbers of transcripts continuously decreased during development until the formation of merozoites. In contrast, transcripts of another microneme encoding gene, *EtMIC5*, were maintained at a higher level during endogenous development.

Transgenesis in *Eimeria* spp. is now routinely used and most studies have focused on the use of *Eimeria* as a vaccine vehicle to deliver foreign antigens [15, 17, 31]. We now report the use of this technology as a convenient and rapid tool for the study of endogenous protein localization; being able to visualise the location of overexpressed proteins fused to a fluorescent reporter has allowed a previously uncharacterised MAR containing protein to be tentatively assigned to the microneme organelle. This is a promising start to further approaches to study gene functionality or use them as “bait” to capture interacting and proximal proteins, as has been recently done in *Toxoplasma* [32, 33].

## Conclusions

The threonine (T) in the HxT motif of Type I MAR domains of *E. tenella* MCPs appears to play an important role in the binding properties of these proteins, as has been suggested before [21], since other variants (HxS and YxE) did not show binding to fetuin or MDBK cells. EtMCPs are expected to be localised in the micronemes, with the aim to confirm this; EtMCP2 was expressed in transgenic *Eimeria* as a fusion with the fluorescent reporter mCherry exhibiting an apical localisation, typical of microneme proteins (and identical to the EtMIC2-mCherry fusion). In addition, EtMCP2 showed a similar pattern of transcripts than other miconeme proteins (EtMIC2 and EtMIC5). We concluded therefore, that at least one of these newly described EtMCPs is located in the micronemes, as was expected.

## Additional file

Additional file 1:
**Table S1.** Primers used in the study. **Figure S1.** Plasmid construct developed and used for the genetic complementation of *E. tenella.* The plasmids contain a double cassette: *mCitrine* - green arrow - flanked by 5*′EtMIC1* and *3′EtMIC1* - green arrows - and EtMIC2 and EtMCP2 fused to *mCherry* - yellow arrow + red arrow - flanked by 5*′EtMIC2* and 3*′EtMIC3* - final purple arrows. (PDF 273 kb)

## Abbreviations

BSA: bovine serum albumin
cDNA: complementary DNA
FACS: fluorescence-activated cell sorting
MAR: microneme adhesive repeat
MCP: MAR containing proteins
MDBK: Madin-Darby bovine kidney
MIC: microneme secretory organelles
PBS: phosphate buffered saline
qPCR: quantitative PCR
REMI: restriction enzyme-mediated integration
RT-qPCR: reverse transcription qPCR
TBS-Tween: tris buffered saline-Tween20
