# Supplementary material for: Characterization of novel microneme adhesive repeats (MAR) in Eimeria tenella
Source: Parasit Vectors. 2017 Oct 17;10:491. doi: 10.1186/s13071-017-2454-4 (PMC5646145; doi:10.1186/s13071-017-2454-4)
Supplement: Additional file 1: — Table S1. Primers used in the study. Figure S1. Plasmid construct developed and used for the genetic complementation of E. tenella. The plasmids contain a double cassette: mCitrine - green arrow - flanked by 5′EtMIC1 and 3′EtMIC1 - green arrows - and EtMIC2 and EtMCP2 fused to mCherry - yellow arrow + red arrow - flanked by 5′EtMIC2 and 3′EtMIC3 - final purple arrows. (PDF 273 kb) [file 13071_2017_2454_MOESM1_ESM.pdf]

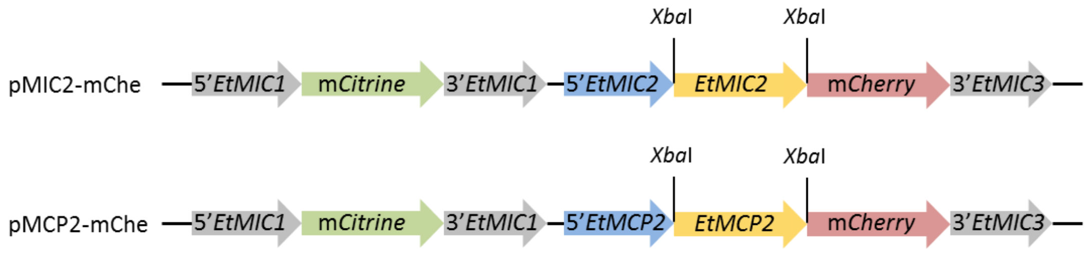

Figure S1. Plasmid construct developed and used for the genetic complementation of *E. tenella*. The plasmids contain a double cassette: *mCitrine* – green arrow – flanked by 5*'EtMIC1* and *3'EtMIC1* – green arrows – and EtMIC2 and EtMCP2 fused to *mCherry* – yellow arrow + red arrow – flanked by 5*'EtMIC2* and 3*'EtMIC3* – final purple arrows –.

Table S1. Primers used in the study

**Name**	**Sequence**
**Expression in *E. tenella***	
Fw-MIC9-XbaI	CCTCTAGAAAGTTTTTCGCGAGCC
Rv-MIC9-XbaI	CGTCTAGACCTCTGTAACGCAACG
**Expression as recombinant proteins**	
Fw-MCP2.1-NcoI	GACCATGGCAGACAAATGCCGTGAGCA
Rv-MCP2.1-NotI	TTGCGGCCGCCTTAGCGTGCCTAAT
Fw-MCP3.3-NcoI	GACCATGGCAAAAGAATCCGGGAGTCTGC
Rv-MCP3.3-NotI	TTGCGGCCGCTGCTGCGACGTTGC
Fw-MCP4.2-NcoI	GACCATGGCAGACAAATGCCGTGAGCA
Rv-MCP4.2-NotI	TTGCGGCCGCCTTAGCGTGCCTAAT
Fw-MCP4.3-NcoI	TGCCATGGCAGCTGGACAGACCTATGAGC
Rv-MCP4.3-NotI	TTGCGGCCGCGCCATGCAGGCATTT
Fw-MCP5.2-NcoI	TGCCATGGCAGCGGCGGCCAGCGGGCT
Rv-MCP5.2-NotI	TTGCGGCCGCGCACATAGCGTCCGT
